# A rare case of a neonate with fallot-type absent pulmonary valve and occlusion of the left main bronchus

**DOI:** 10.1186/s13019-024-02534-z

**Published:** 2024-02-06

**Authors:** Chunjie Mu, Minzhang Zhao, Runwei Ma, Xiang Li, Min Liu, Yao Deng

**Affiliations:** 1 Department of Cardiac Surgery, Fuwai YunnanHospital, Chinese Academy of Medical Sciences, 528, Shahe North Road, Wuhua District, Kunming, Yunnan Province China; 2 Department of Cardiology, Sichuan University West China Hospital, Chengdu, China

**Keywords:** Tetralogy of Fallot, Absent pulmonary valve, Occlusion of the left main bronchus

## Abstract

Fallot-type absent pulmonary valve is a rare and complex congenital heart disease. Repair surgery for this condition during the neonatal period has a mortality rate of over 50%. We reported a neonate with Fallot-type absent pulmonary valve and occlusion of the left main bronchus. The patient’s pulmonary artery had unusual anatomy of a type that has not previously been reported. This case report outlines a successful treatment strategy for patients with complex congenital heart disease and airway occlusion during the neonatal period and the effect of these unusual anatomical conditions on postoperative outcomes.

**Supplementary Information:**

The online version contains supplementary material available at 10.1186/s13019-024-02534-z.

## Introduction

Absent pulmonary valve of the type associated with tetralogy of Fallot (Fallot-type absent pulmonary valve, or TOF-APV) is a rare and complex congenital heart disease. Repair surgery for TOF-APV during the neonatal period has a mortality rate of over 50% [1, 2]. We reported a neonate with TOF-APV and occlusion of the left main bronchus. The patient’s pulmonary artery (PA) had unusual anatomy of a type that has not previously been reported. The surgery was performed successfully at 27 days old with a targeted surgical plan, and the patient was discharged 21 days after the surgery.

## Case report

**Fig. 1:**
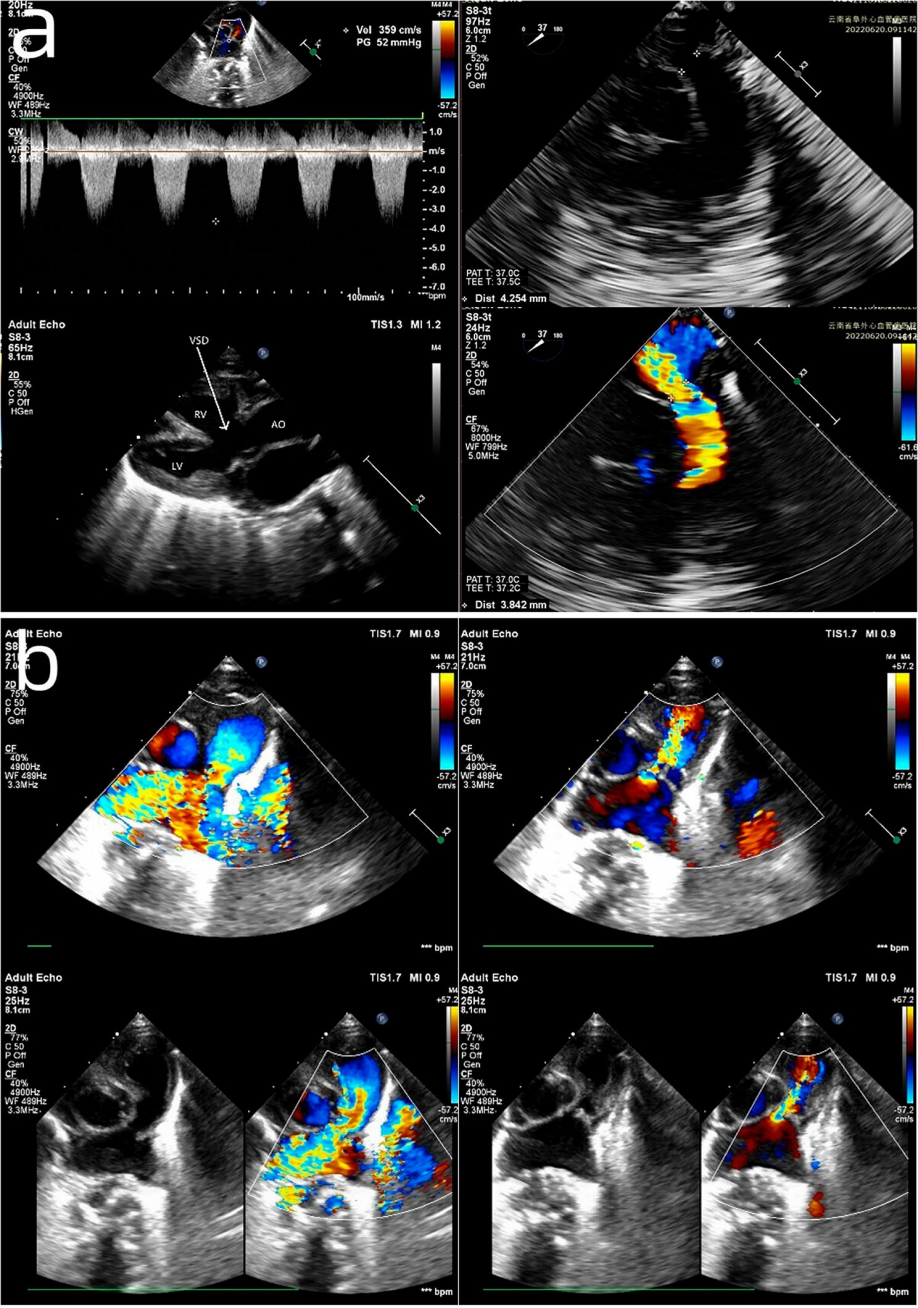
Preoperative ultrasound showed massive pulmonary artery regurgitation (**a**), stenosis and aneurysmal dilatation of the main pulmonary artery (**b**), ventricular septal defect (VSD), and aorta (AO) overriding. LV, left ventricle; RV, right ventricle

**Fig. 2:**
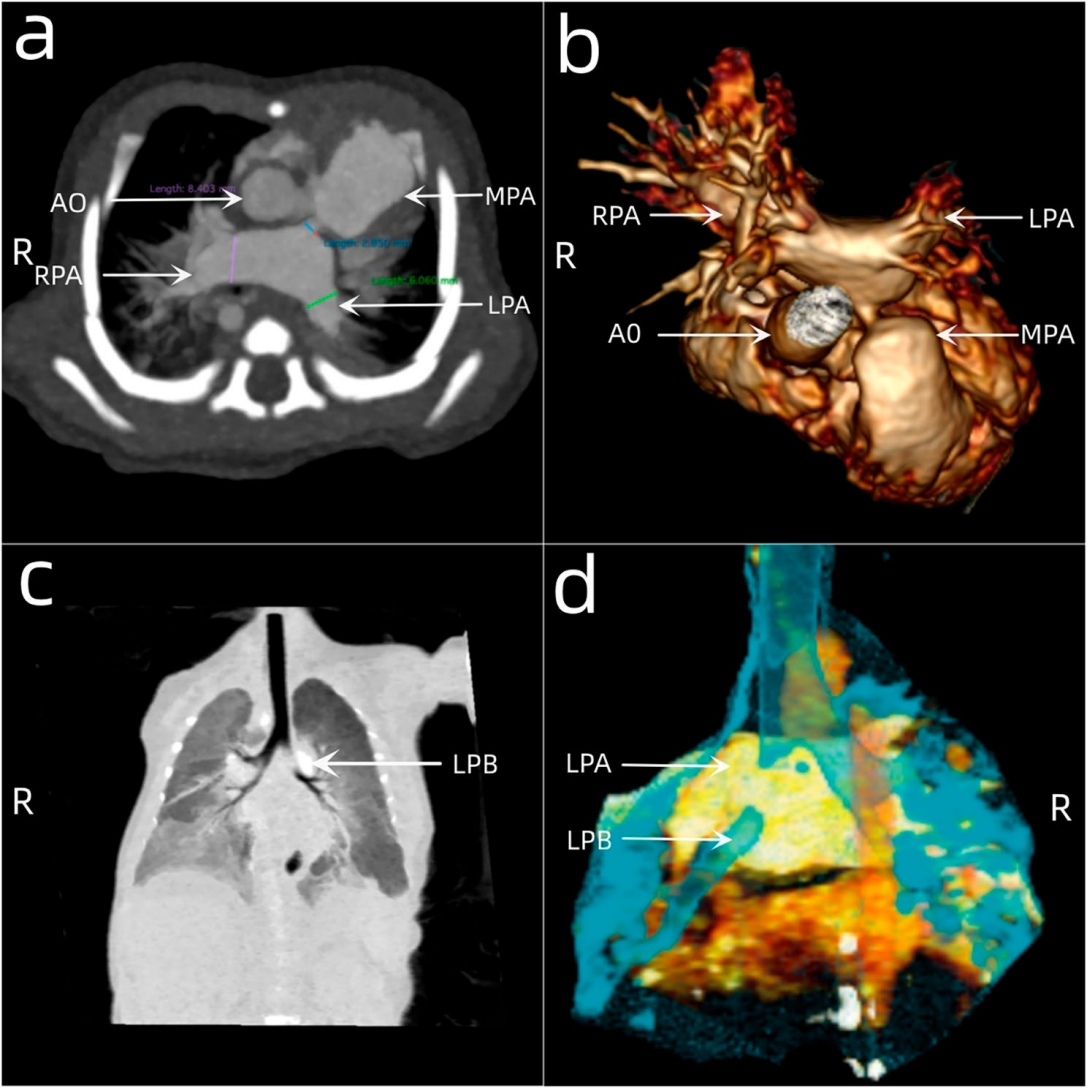
Preoperative enhanced computed tomography of the heart shows dilation in the proximal and stenosis in the distal segments of the main pulmonary artery, extreme aneurysmal expansion of the left and right pulmonary arteries, and occlusion of the left main bronchus. AO, aorta; MPA, main pulmonary artery; RPA, right pulmonary artery; LPA, left pulmonary artery; LPB, left principal bronchus

A baby with TOF-APV was born at 36 weeks of gestation, weighing 2.4 kg. The parents took her to the paediatrics department for endotracheal intubation because of asphyxia just after birth. The baby was transferred to the paediatric intensive care unit (PICU) of our hospital at 13 days old. Arterial blood gas analysis showed that the saturation of oxygen was 44.6% and that the partial pressure of oxygen was 33.9 mmHg. Echocardiography (Fig. 1) revealed an enlarged right heart, a ventricular septal defect with a diameter of 9 mm, a bidirectional shunt at the ventricular level, right ventricular outflow tract obstruction due to muscular hypertrophy, aortic widening and overriding, an atrial septal defect with a diameter of 2 mm, a patent ductus arteriosus with a diameter of 1.5 mm, and an absent pulmonary valve. The pulmonary valve annulus had a diameter of 4.9 mm, there was substantial pulmonary regurgitation and aneurysmal dilatation of the PA, the main pulmonary artery (MPA) was severely stenotic caudally with a PA systolic flow rate of 4.2 m/s, a maximum pressure difference of 71 mm Hg and a diastolic flow rate of 2.3 m/s, indicating a pressure difference of 21 mm Hg, and the right aortic arch had mirror-image branching. Furthermore, enhanced computed tomography (CT) examination of the heart showed dilation in the proximal segments and stenosis in the distal segments of the MPA, severe stenosis and occlusion of the left main bronchus (Fig. 2), and multiple sites of exudation and consolidation in both lungs.

After admission, the patient was placed on a non-invasive ventilator with 40% oxygen to assist with breathing. Under ventilator assistance, the patient’s oxygen saturation was 71.4–82.8%. Because the patient had severe stenosis of the left bronchus, we gave anti-microbial prophylaxis, as well as adequate nutritional support and nursing care; however, non remitting wet rales in left lung suggested that the patient could not be removed from the noninvasive ventilator. When the patient was 27 days old, surgery was performed under general anaesthesia with hypothermic cardiopulmonary bypass. During the surgery, we observed a large amount of connective tissue adhering to the posterior side of the left pulmonary artery (LPA), and the caudal end of the MPA was also surrounded by connective tissue, this abnormality was not observed in the other parts of the PA; additionally, severe focal constriction was observed at the posterior segment, appearing as a clubfoot and showing tissue attachment to the MPa. Pulmonary arterioplasty was performed to treat the dilated PA. We dissociated the adherent LPA and loosened the connective tissue attached to the caudal end of the MPA; cut the anterior walls of the MPA, LPA, and right pulmonary artery (RPA); and sutured the LPA and RPA. Next, bovine pericardial tissue patches of appropriate sizes were sutured to the MPA and the anastomosis of the LPA and RPA.We simultaneously used autologous pericardial slices to reconstruct the pulmonary valve using the monocusp valve. Radical surgery for tetralogy of Fallot (TOF), patent ductus arteriosus closure, and atrial septal defect repair were performed to repair other structural cardiac abnormalities. The surgery was uneventful, and the patient was sent to the PICU postoperatively.

**Fig. 3:**
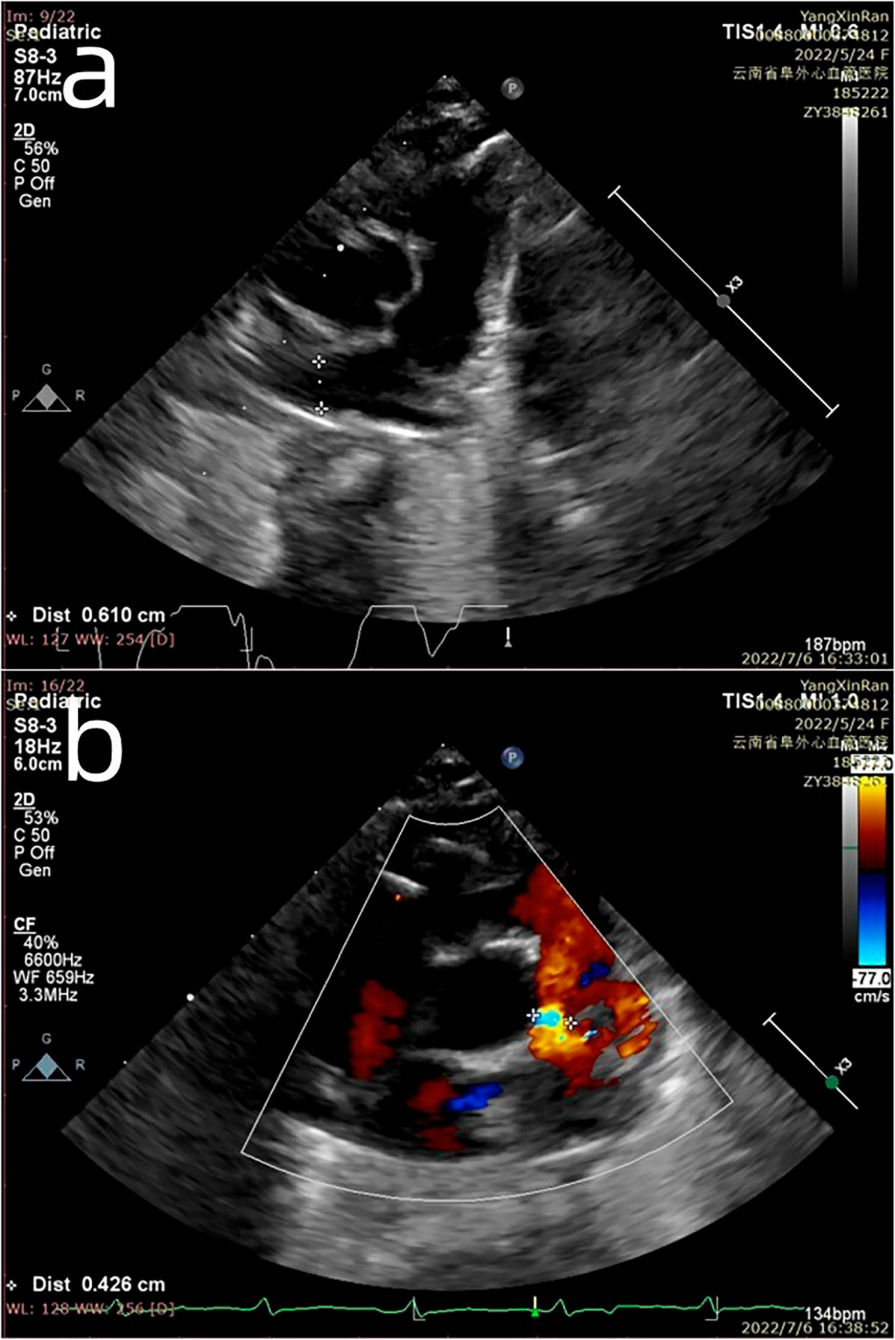
Post-operative ultrasound showed satisfactory pulmonary arterioplasty (**a**) with mild to moderate pulmonary valve regurgitation (**b**)

**Fig. 4:**
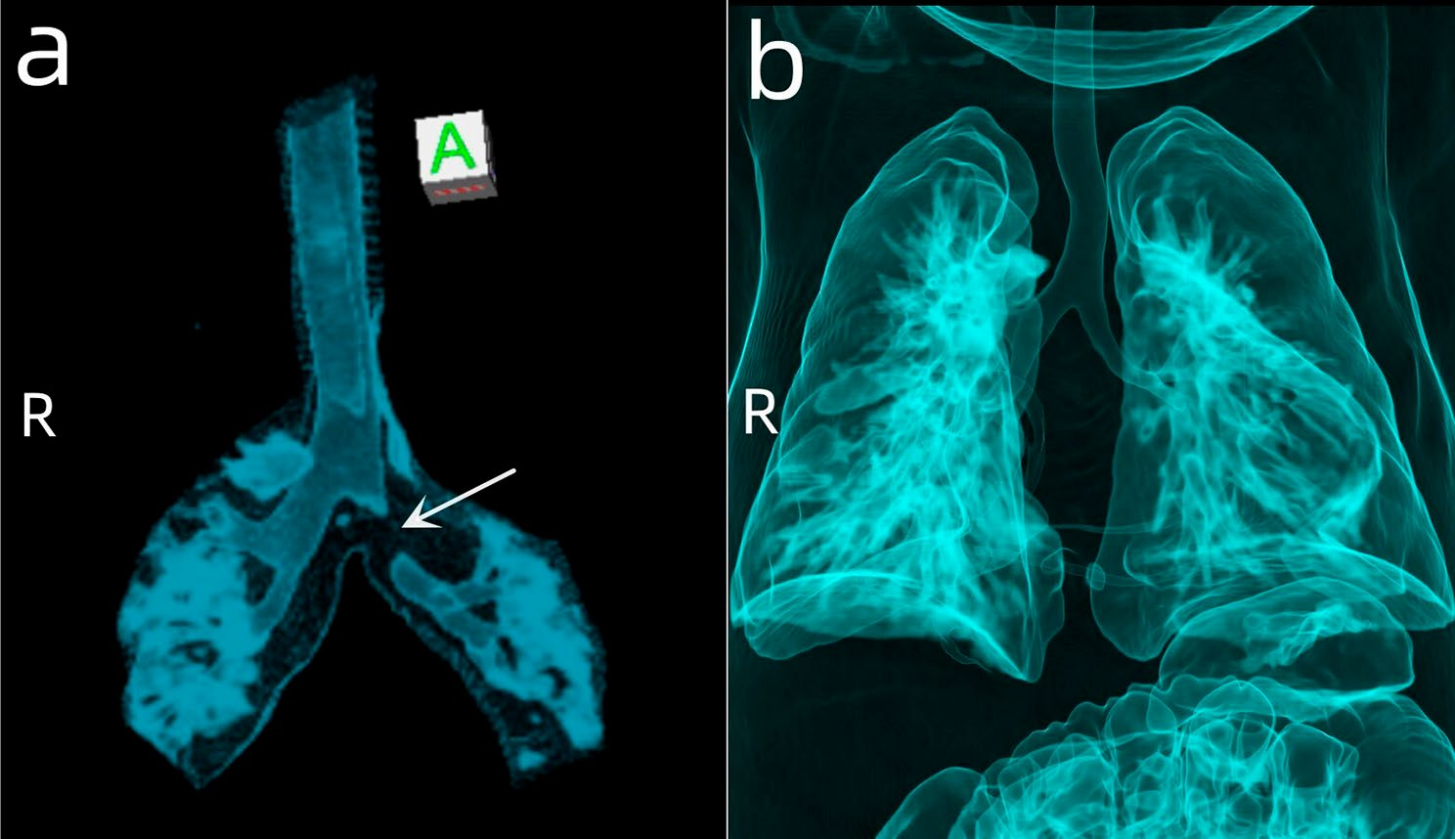
Three-dimensional reconstruction of the CT scan shows that the airway stenosis was relieved after surgery. Before surgery (**A**), after surgery (**B**)

After surgery, the patient received endotracheal intubation; continuous ventilator-assisted treatment; and symptomatic support treatment such as fluid infusion, diuresis, and anti-microbial prophylaxis to promote the recovery of cardiopulmonary function. The patient’s postoperative oxygen saturation was 93.1–98.9%. Two days postoperatively, the patient was switched from endotracheal intubation to a non-invasive ventilator, and oxygen was delivered through a mask 8 days postoperatively. Immediate postoperative echocardiography showed that the inner diameter of each PA (MPA 11 mm, RPA 3.4 mm, and LPA 5 mm) was decreased compared to the preoperative results (MPA 14 mm, RPA 10 mm, and LPA 8 mm). However, the regurgitation of the pulmonary valve did not completely disappear, and the degree was low-moderate, the systolic flow velocity of the pulmonary valve was 1.8 m/s and the differential pressure was 13 mmHg. The patient was transferred from the PICU to the general ward of the cardiac surgery department 14 days postoperatively, the patient returned for echocardiography, and the results were roughly the same as the pre-discharge results, unexpectedly, the diameters of the LPA and RPA increased to 7 mm and 6 mm, respectively, pulmonary valve regurgitation exists(Figure 3). Plain chest CT showed no airway stenosis (Fig. 4). During this period, the patient developed no major complications and the wet rales of the left lung gradually resolved and discharged 21 days postoperatively.

## Discussion

The symptoms of TOF-APV were first described by Chevers in 1847. TOF-APV presents as a missing or undeveloped pulmonary valve with the anatomy of TOF. Due to massive pulmonary valve regurgitation, the total pulmonary blood flow increases, resulting in the expansion of the MPA and PA branches [3]. The dilated PA may compress the bronchus, leading to airway stenosis. Auxiliary examination is a very important diagnostic tool for TOF-APV. Echocardiography can detect massive pulmonary regurgitation and allow a definite diagnosis, and enhanced CT can examine the structure of the heart and the state of the blood vessels and airways. Furthermore, bronchoscopy can evaluate the degree of airway stenosis when necessary [2].

Presently, as is true of most congenital heart diseases, there is no good palliative treatment for TOF-APV, and the timing of surgery depends on the patient’s symptoms. Several studies have shown that preoperative ventilation and neonatal surgery greatly affect postoperative ventilation time and survival rates [4, 5]. Patients who undergo surgery for TOF-APV during the neonatal period have a mortality rate of over 50%, while the mortality rate significantly decreases to less than 20% in infants and children [1, 2]. Therefore, performing surgeries during the neonatal period is not recommended due to the high risk of adverse outcomes. Patients with symptoms, such as asphyxia, should receive symptomatic treatment first and then be prepared for surgery. The case we present is unusual in that the patient’s left main bronchus was compressed, which resulted in severe stenosis, and the left lung showed exudates and solid lesions. We did our best to treat the patient’s symptoms, but the child could not be weaned from mechanical ventilation, so we opted for early surgical intervention.

For the surgical plan, pulmonary arterioplasty, pulmonary valvuloplasty, or complete central pulmonary artery replacement [5] with a valved bovine jugular vein conduit can be performed to treat dilated PA and APV. Although pulmonary valve regurgitation can be repaired more effectively through pulmonary artery replacement than pulmonary arterioplasty and pulmonary valvuloplasty, pulmonary artery replacement may increase the operative time and the need for repeated intervention [4]. Other surgeries can be simultaneously performed to repair structural cardiac abnormalities, such as TOF.

Unusual anatomical structures may have unexpected effects on patients and cause unexplained symptoms. In this case, the patient had an anatomically unusual type of PA with a dilated MPA and severe stenosis at the end of the MPA surrounded by connective tissue, which further led to severe dilatation of the LPA and RPA after stenosis, a combination that has not been previously reported in the literature. However, under the premise that symptomatic treatment was not effective, we chose an aggressive surgical intervention, in which conventional methods were applied to correct TOF while closing the VSD and PDA. With respect to the APV, because the pulmonary valve annulus was small, we applied the monocusp valve technique. We loosened the connective tissue that caused adhesion of the LPA to the left main bronchus and created stenosis caudal to the MPa, and reshaped the PA where there was dilatation and stenosis to relieve the extrusion caused by LPA dilatation to the left bronchus.

We concluded that the three causes of asphyxia were as follows. First, the dilated LPA compressed the left main bronchus. This occurrence is a common cause of airway stenosis in patients with TOF-APV; however, not all patients present with airway occlusion. Second, the anatomically unusual stenosis in the distal segment of the MPA altered the stress relationship between the PA and airway, causing severe pressure on the left main bronchus. Third, the connective tissue adhered to the posterior side of the LPA. Therefore, we formulated a targeted surgical plan for the patient and achieved a satisfactory result.

In conclusion, we report the successful surgical management of TOF-APV and airway occlusion during the neonatal period.

### Electronic supplementary material

Below is the link to the electronic supplementary material.

Video 1: Three-dimensional reconstruction of the heart

Video 2: Three-dimensional reconstruction of the heart and airway

Video 3: Pre-operative echocardiography

Video 4: Pre-operative echocardiography

Video 5: Pre-operative echocardiography

Video 6: Post-operative echocardiography

## Glossary of abbreviations

TOF-APV: Fallot-type absent pulmonary valve
PA: Pulmonary artery
PICU: Paediatric intensive care unit
CT: Computed tomography
MPA: Main pulmonary artery
LPA: Left pulmonary artery
RPA: Right pulmonary artery
TOF: Tetralogy of Fallot
